# Values of ultrasound features and MMP-9 of papillary thyroid carcinoma in predicting cervical lymph node metastases

**DOI:** 10.1038/s41598-017-07118-7

**Published:** 2017-07-27

**Authors:** Yan Zhang, Yu-kun Luo, Ming-bo Zhang, Jie Li, Chang-tian Li, Jie Tang, Jun-lai Li

**Affiliations:** 1 Departments of Ultrasound, Chinese People’s Liberation Army General Hospital, Beijing, China; 2 Departments of Pathology, Chinese People’s Liberation Army General Hospital, Beijing, China

## Abstract

Preoperative assessment of the cervical lymph node status is important in therapeutic schedule and further evaluations of prognosis for papillary thyroid carcinoma (PTC) patients. Our aim was to investigate the diagnostic values of conventional ultrasound (US), contrast-enhanced ultrasound (CEUS) features and the expression of MMP-9 of PTC in predicting the cervical lymph node metastases (LNM). In total, 156 patients with PTC confirmed by surgical pathology were included. Seventy-one patients had cervical LNM, while 85 patients had no LNM. The patients had cervical LNM (39.51 ± 13.29 years) were younger than those had no LNM (44.15 ± 10.94 years) (*P* = 0.02). Multivariate logistic regression results showed that tumor size ≥0.95 cm (OR = 13.47), ill-defined margin (OR = 4.31), internal heterogeneous low-enhancement (OR = 5.19) and ECE (OR = 25.25) were predictive for the presence of cervical LNM. The detection rate of ECE for the PTC with LNM by CEUS (81.48%, 44/54) was higher than by US (46.30%, 25/54). There was significant difference in MMP-9 intensity between PTC with and without cervical LNM (*P* = 0.000), and intense reactions (+++) were mainly found in the PTCs with LNM (80.95%, 17/21). In conclusion, the combination of conventional US, CEUS features and MMP-9 expression may serve as an effective tool for predicting the cervical LNM of PTC.

## Introduction

The incidence of thyroid cancer (TC) has been greatly increasing around the world due to the early detection by ultrasound, fine-needle aspiration (FNA) and core-needle biopsy (CNB). Papillary thyroid carcinoma (PTC) is the most common type of TC with an excellent prognosis. However, the clinical behaviors of this cancer are complex and varied. Cervical lymph node metastasis (LNM) is frequently observed in PTC patients with an average incidence of 60%^1^ because PTC tends to spread via lymphatic ducts^2^. According to some studies, PTC LNM are reported to have no significantly clinical effects on the outcomes in low-risk patients, however, a study with large samples (9904 patients) found that PTC with LNM, age >45 years, distant metastasis, and large tumor size could significantly predict poor overall survival outcome^3^. Other studies also reported that LNM was associated with increased local recurrence, and even decreased survival in some high-risk groups^4–6^. Both ATA guideline^7^ and Chinese guideline^8^ for diagnosis and treatments of thyroid nodules and differentiated TC recommend subtotal or total thyroidectomy as the main surgical procedures for differentiated TC, and the therapeutic or prophylactic lymph node dissection are also included. In several studies, prophylactic dissection has shown no improvement in long-term outcome, but increasing the likelihood of temporary morbidity because of the large surgical wound including hypocalcemia, laryngeal recurrent nerve injury, and so on. Therefore, preoperative assessment of the cervical LN status is important in therapeutic schedule and further evaluation of prognosis for PTC patients. However, some minimal LNM may be difficult to be detected, which may not show any abnormal findings in preoperative US scanning^9, 10^ and was found to be common in postoperatively pathological examinations. The requirement for preoperative predictions of the cervical LNM by investigating the TC characteristics is urgent.

Conventional high resolution ultrasound (US) had good diagnostic sensitivity and specificity in the preoperative diagnosis of TC and following-up investigations^11^, but limitations still exist in the assessment of microvessel supply in tumor, which was associated with aggressive behaviors of cancer. Contrast enhanced ultrasonography (CEUS) is a promising tool in investigating tissue microvessel perfusion and its internal enhancement intensity and patterns have been reported to improve the identification of TC when comparing with conventional US^12–14^. In addition, a recent study^15^ found the peripheral ring enhancement pattern on CEUS also was an important parameter for differential diagnosis of thyroid nodules, and irregular peripheral rings could predict the malignancy. CEUS provides additional information of TC, and combining with US can reduce the unnecessary biopsies. Whether the US and CEUS features of PTC could predict the risk of cervical LNM should be investigated.

In cancer research, matrix metalloproteinases (MMPs), a family of zinc-dependent endopeptidases with the capacity to degrade extracellular matrix proteins and basement membranes^16^, is proved to play an important role in multiple stages of cancer progression and metastasis. Among the MMPs, a subset called gelatinase, consisting of MMP-2 (gelatinase A) and MMP-9 (gelatinase B), has gained the most attention of studies on the acquisition of invasive and metastatic tumor properties, as they could degrade collagen IV which is the major component of the basement membrane^17, 18^. In thyroid diseases, much evidence^19, 20^ demonstrated that MMP-9 is overexpressed in TC, especially in PTC, when compared to the benign tumor and normal tissues, and the active MMP-9 contributes to the development and metastasis of TC.

In this study, we will investigate the relationship between cervical LNM and US, CEUS features and MMP-9 expressions of the PTC.

## Methods

### Patients

The study, which was approved by General Hospital of Chinese People Liberation Army Research Ethics Committees, was carried out in this hospital. All methods were performed in accordance with the institutional guidelines and regulations, and written informed consents were obtained from all subjects included in the study. From December 2014 to January 2016, 156 inpatients with suspicious thyroid lesions scheduled for surgery underwent conventional US and CEUS examination preoperatively. Exclusion criteria were: (1) Patients allergic to sulfur hexafluoride microbubbles (SonoVue). (2) Patients with benign tumor or other types of thyroid carcinoma; (2) Patients underwent thyroid nodule minimally invasive ablation, any cervical surgery, chemical therapy or radiotherapy. The mean ± SD age of 156 patients were 42.04 ± 12.248 years (range, 14–78 years). The mean nodule maximum diameter was 1.117 ± 0.856 cm (range, 0.3 cm–6.8 cm).

Hemithyroidectomy with ipsilateral and central LN dissection was performed in 55 patients, and hemithyroidectomy with only central LN dissection was performed in 81 patients. Total thyroidectomy with bilateral and central LN dissection was performed in 20 patients with clinically suspicious LNM or bilateral lesions.

### Ultrasound examination

L12-5 probe of Philips iU22 US system (Philips, Amsterdam, Holland) were used to perform US examination with frequency of 9–12 MHz.

Patients were scanned in the supine position with their neck extended. The size, position, boundaries, internal structure and blood supply of the lesions were assessed. If more than 3 nodules with suspicion of malignancy were found in thyroid gland, then the maximum diameter of the largest lesion was record and included in the data analysis. The vascular pattern on color Doppler flow imaging (CDFI) was classified according to Rago^21^ as follows: Type I, absence of blood flow; Type II, perinodular blood flow; and Type III, marked intranodular blood flow.

### CEUS examination

L9-4 probe of Philips iU22 US system (Philips, Amsterdam, Holland) were used to perform US examination with frequency of 7 MHz and a mechanical index (MI) of 0.08. The contrast agent used in this study was 59 mg dry powder SonoVue (Bracco S.p.A Inc., Milan, Italy) and was made up in 5 ml of normal saline, which was administered intravenously at the elbow. The dose used was 2.0 ml for each CEUS examination.

The real-time microbubble perfusion within lesions and surrounding tissues were observed for a minimum of two minutes and recorded on the ultrasound machine’s internal hard drive.

### Immumohistochemistry

Forty-two paraffin sections of resected PTCs including 21 PTC from patients with LNM and 21 without LNM were selected randomly to stain for MMP-9. The sections with thickness of 4 μm were obtained. An anti-MMP-9 antibody was used to stain MMP-9 protein, and the second antibody used Biotinylated goat- anti-mouse antibody (Santa Cruz, USA, 1:100). The signal was enhanced with the avidin-biotin-peroxidase complex followed by visualization of the reaction with 3,3′-diaminobenzidine tetrahydrochloride (DAB) solution (Peroxidase Substrate Kit, Vector Laboratories, Burlingame, CA, USA). The slides were counterstained with hematoxylin and examined using an Axio Imager 1.0 microscope (Carl Zeiss, Jena, Germany) with a Nikon DS-U3 Digital Camera System (Tokyo, Japan). Tan nuclear stainning was considered to be positive.

### Image interpretation and analysis

The recorded CEUS images were reviewed and the enhanced intensities in thyroid nodules were classified as: no enhancement; low enhancement; iso-enhancement and hyper-enhancement. When enhancement was present, it was further assessed for the degree of homogeneity^22, 23^, and producing the following lesion enhancement categories: (1) homogeneous low-enhancement; (2) heterogeneous low-enhancement; (3) homogeneous iso-enhancement; (4) heterogeneous iso-enhancement; (5) homogeneous hyper-enhancement; (6) heterogeneous hyper-enhancement.

The peripheral ring appearances of nodules were divided into: (1) regular high-enhancement ring; (2) irregular high-enhancement ring; (3) regular no-enhancement ring; (4) irregular no-enhancement ring. Regular rings were defined as round or oval, and their thickness was uniform. On the contrary, irregular rings were misshapen, and their thickness was non-uniform.

The extracapsular extension (ECE) on the US image were defined that the anterior and posterior hyperechoic thyroid capsular were discontinued. During the real-time and multi-angle scanning, the ECE on CEUS was shown as a low or non-enhancing area on the thyroid capsular which is invaded by malignant nodules^24^.

Two US reviewers, both of them did not perform any examinations in this study, viewed US and CEUS images independently. These two reviewers were specialized in the diagnosis of thyroid disease and had more than 10 years of experience in thyroid US and CEUS.

The staining for active MMP-9 in tumor tissue was graded as follows according to the staining intensity and distribution of immunoreactivity within a single tissue section^25^: (0) no reaction (−); (1) weak reaction (+); (2) moderate reaction (++); (3) intense reaction (+++). Immunohitochemical staining results was evaluated by two independent observers, with high concordance. When there was doubt between two scores, the observers reexamined the section together in order to establish a consensus.

### Statistical analysis

All statistical analyses were performed using the Statistical Package for the Social Sciences (SPSS) software package, Version 13 for Windows (SPSS Inc., Chicago, IL, USA). The Pearson chi-squared test (*χ* ^2^) and Fisher’s exact test were used to compare the categorical data. Quantitative data were presented as mean ± SD, and the *t*-test was used for comparison. Receiver operating characteristic curves (ROC) studies were drawn to determine the cutoff value for the tumor size. Mann-Whitney U was applied for comparing the continuous variables depending on the normality of the distribution. All ultrasound features that proved to be statistically significant on univariate analysis were analyzed to assess independent association with cervical LNM using multivariate logistic regression. *P* < 0.05 was considered statistically significant.

## Results

### Surgical pathologic results

**Figure 1:**
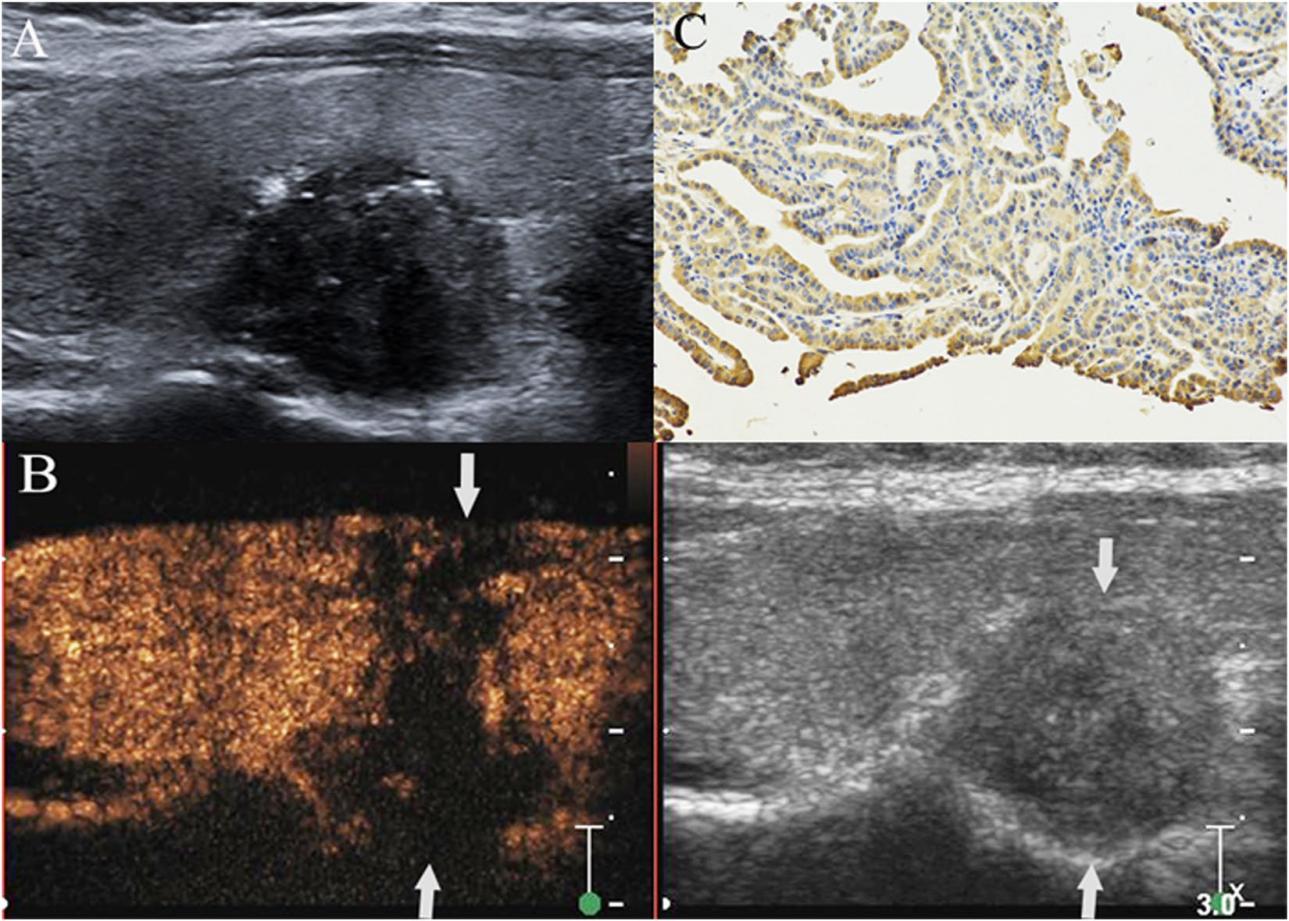
PTC with cervical LNM. A hypoechoic nodule with ill-defined margin, irregular shape and some microcalcifications was found at the lower pole of the right thyroid lobe in a 34- year– old female patient (**A**). The thyroid focal posterior capsular was bulged by nodule. The size of nodule was 1.6 cm × 1.5 cm × 1.0 cm. CEUS showed that a heterogeneous low-enhancement in nodule (left image) and its area (1.8 cm × 1.6 cm × 1.2 cm) was larger than the nodule region displayed on grey-scale image (right image) (arrow). Both the anterior and posterior thyroid capsular were broken and invaded by the nodule (arrows) (**B**). Intense expression (+++) (arrow) of matrix metalloproteinase-9 was found in the cytoplasm of the malignant cells of a PTC sample, as shown by the brown staining (magnification, ×200) (**C**). Surgical results: PTC with ipsilateral region III-V and central compartment region LNM.

**Figure 2:**
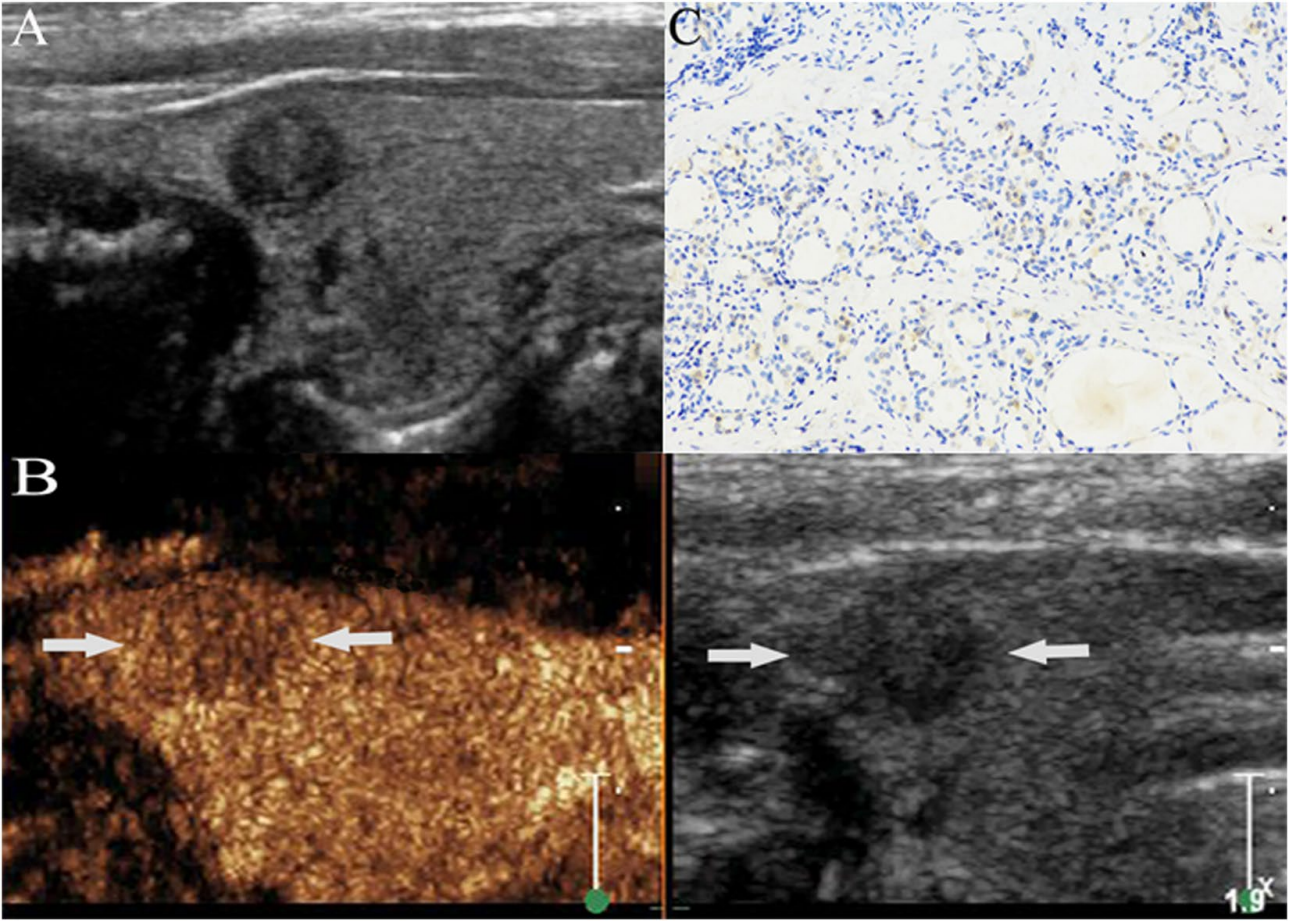
PTC without cervical LNM. A hypoechoic nodule with ill-defined margin and regular shape was found at the upper pole of the right thyroid lobe in a 44-year-old male PTC patient (**A**). The size of nodule was 0.6 cm × 0.5 cm × 0.6 cm. CEUS showed a slight low-enhancement in the nodule (arrow), and the thyroid capsule was intact (**B**). Weak expression (+) (arrow) of matrix metalloproteinase-9 was found in the cytoplasm of the malignant cells of a PTC sample, as shown by the brown staining (arrow) (magnification, ×200) (**C**). Surgical results: PTC without cervical LNM.

Postoperative pathological results revealed that 71 of 156 patients who suffered from PTC had cervical LNM (Fig. 1). Among these patients, only central compartment LNM were found in 24 patients, ipsilateral cervical (region II-V) LNM were found in 19 patients, ipsilateral and central compartment LNM were found in 18 patients, contralateral cervical (region III-V) and central compartment LNM were found in one patients. Both lateral cervical and central compartment LNM were found in 9 patients. ECE was found by pathology in 54 PTCs. Eighty-five patients had no cervical LNM (Fig. 2), and no thyroid capsule was broken by tumor.

### Patient clinical characteristics

The clinical characteristics of the patients were shown in Table 1. There was significant difference in patients’ ages between PTC with and without cervical LNM (*P* = 0.02), while there were no significant differences in the gender of patients and multifocality of PTC between the two groups (*P* = 0.83, 0.85).

**Table 1:** Clinical characteristics of patients with or without cervical LNM.

characteristics	metastases (n = 71)	No metastases (n = 85)	*t*(*χ* ^2^)	*P*
Age (years)	39.51 ± 13.29	44.15 ± 10.94	2.39	0.02
Gender			0.05	0.83
Male	18	17	0.64	0.42
Female	53	68		
Multifocality			0.04	0.85
yes	24	30		
no	47	55		

LNM: lymph node metastases.

### Conventional US and CEUS characteristics

Conventional US and CEUS features of PTC with LNM or not were showed in Table 2.

**Table 2:** Ultrasonographic nodule characteristics with or without cervical LNM.

Parameters	Characteristics	Metastasis		*X* ^*2*^(*t*, *U*)	*P* Value
		Yes (n = 71)	No (n = 85)		
Size, cm		1.473 ± 1.085	0.819 ± 0.418	63.590	0.000
Shape	Regular	4	21	10.457	0.0012
	Irregular	67	64		
Margin	Well-defined	16	42	11.965	0.0005
	Ill-defined	55	43		
Echogenicity	Homogeneous	53	80	11.667	0.0006
	Heterogeneous	18	5		
	Hypoechoic	68	81	0.060	0.807
	Heterogeneous isoechoic	2	2	0.106	0.744
	Cystic-solid mixed	1	2	0.025	0.875
Calcification	Absent	23	45	6.642	0.01
	macrocalcification	6	7	0.002	0.961
	Microcalcification	42	33	6.406	0.011
Vasculalarity	Type I	32	45	2695.00	0.216
	Type II	25	30		
	Type III	14	10		
Internal enhancement pattern	Homogeneous low-enhancement	4	22	11.420	0.0007
	Heterogeneous low-enhancement	51	37	12.602	0.0004
	Homogeneous isoenhancement	4	7	0.3995	0.527
	Heterogeneous isoenhancement	4	10	1.780	0.182
	Homogeneous high-enhancement	1	3	0.697	0.404
	Heterogeneous high-enhancement	7	6	0.397	0.529
Peripheral enhancement rings	No ring	58	66	0.388	0.533
	Irregular high-enhanced rings	2	11	2.167	0.141
	Irregular non-enhanced rings	10	13	4.25	0.04
	Regular non-enhanced rings	0	3	1.44	0.23
ECE on CEUS	Yes	44	4	59.557	0.0000
	No	27	81		
ECE on US	Yes	25	1	32.265	0.0000
	No	46	84		

LNM: lymph node metastases.

CEUS: contrast-enhanced ultrasound.

US: ultrasound.

ECE: extracapsular extension.

**Figure 3:**
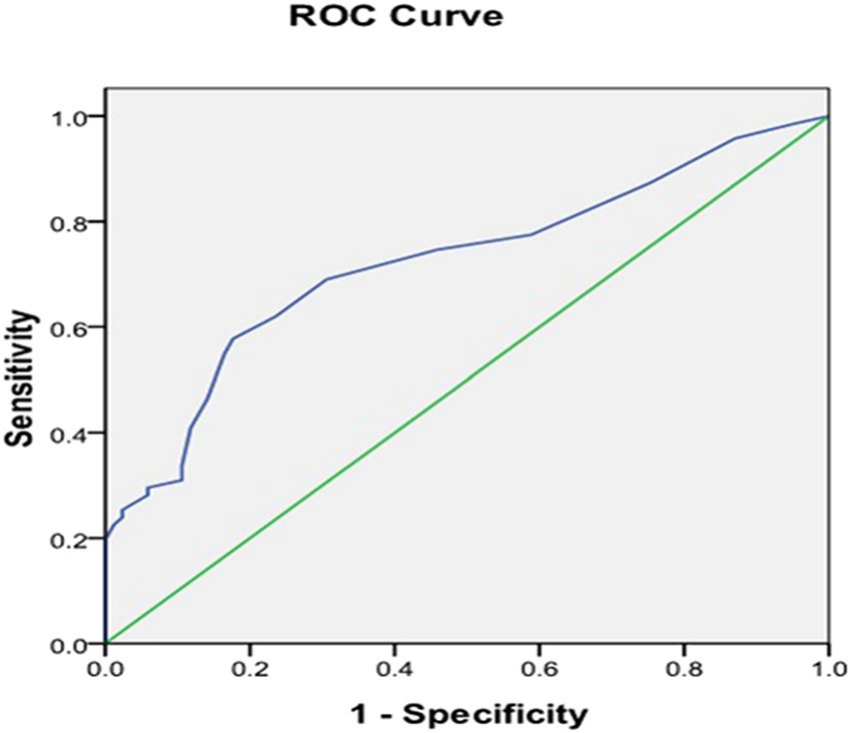
Receiver-operating Characteristic (ROC) curve of nodule size for predicting the risk of cervical LNM. Blue line shows an ROC plot for nodule size with an AUC of 0.72 (95% CI: 0.64–0.80).

Maximum diameters of PTCs (1.47 ± 1.09 cm) with LNM were larger than those without LNM (0.82 ± 0.42 cm) (*P* = 0.000), and its optimal cut-off value of tumor size (0.95 cm) yielded the highest sensitivity (62%) and specificity (76.5%) with an area under curve (AUC) of 0.72 (95% CI: 0.64–0.80) for discriminating between PTC with cervical LNM or not (Fig. 3).

PTCs with LNM were displayed with tumor size ≥0.95 (*P* = 0.000), ill-defined margin (*P* = 0.0005), irregular shape (*P* = 0.001), heterogeneous echogenicity (*p* = 0.0006), present calcification (*P* = 0.01), especially microcalcification (*P* = 0.01), and ECE (*P* = 0.000) on gray US imaging, meanwhile they were displayed with internal heterogeneous low-enhancement pattern (*P* = 0.0004), peripheral irregular no-enhancement rings (*P* = 0.04), and ECE (*P* = 0.000) on CEUS.

Multivariate analyses showed (Table 3) that tumor size (OR = 13.47), margin (OR = 4.31) heterogeneous low-enhancement (OR = 5.19) and ETE (OR = 25.25) on CEUS were associated with cervical LNM (Fig. 1). ROC analyses showed that the overall diagnostic performance of ECE on CEUS (AUC: 0.786, 95% CIs: 0.710–0.863) was superior to other predictors (Table 4), and its diagnostic sensitivity and specificity for PTC with cervical LNM were 62.0% and 95.3%, respectively.

**Table 3:** Multivariate analysis of association of cervical LNM and ultrasonographic characteristics.

parameters	*P* value	OR	95% CI (lower)	95% CI (upper)
Tumor size	0.000	13.471	2.126	11.977
Margin	0.038	4.311	1.060	7.562
Heterogeneous low-enhancement	0.023	5.190	1.175	8.519
ECE on CEUS	0.000	25.250	6.367	67.948

LNM: lymph node metastases.

ECE: extracapsular extension.

**Table 4:** ROC analyses of the independent parameters in identifying cervical LNM from patients with PTCs.

Parameters	AUC	95% CI	Cut-off value	Sensitivity	Specificity
Tumor size	0.722	0.640–0.804	0.95 cm	62.0%	76.5%
Margin	0.634	0.547–0.722	Ill-defined	77.5%	49.4%
Heterogeneous low-enhancement	0.642	0.554–0.729	Heterogeneous low-enhancement	71.8%	56.5%
ECE on CEUS	0.786	0.710–0.863	ECE	62.0%	95.3%

LNM: lymph node metastases.

ECE: extracapsular extension.

AUC: area under curve.

The detection rate of ECE for the PTC with LNM by CEUS (81.48%, 44/54) was higher than by US (46.30%, 25/54).

### MMP-9 expression results in PTCs from patients with and without LNM

The results of immunohistochemical staining for MMP-9 was showed in Table 5. MMP-9 was expressed in all PTCs. There is significant difference in MMP-9 intensity between PTC with and without cervical LNM (u = 90.5, *P* = 0.000), and intense reactions (+++) were mainly found in the PTCs with LNM (80.95%, 17/21).

**Table 5:** MMP-9 expression in PTC with and without cervical LNM.

Pathology	+	++	+++	Total	U	*P*
No metastasis	1	16	4	21	90.5	0.000
Metastasis	1	3	17	21		

PTC: papillary thyroid carcinoma.

LNM: lymph node metastases.

## Discussion

The clinical characteristics of patient with cervical LNM from PTC has been investigated and reported in many researches, but the results were on controversy. When the incidence is increasing worldwide, more and more young patients with PTC were detected, even in some children and adolescents. According to Siddiqui S *et al*.^20^, the age over 45 years old was an important predictor of LNM, and correlated to the recurrence and survival of patients. In this study, we also found that there was significant difference in patients’ age between the PTC with and without LNM (P = 0.02), and the patients with LNM (39.51 ± 13.29) were younger than those without LNM (44.15 ± 10.94). Therefore, the young patients with suspicious thyroid nodules should be paid more attention to their cervical LN condition. Another study^3^ showed that male gender was one of the predictive factors for presence central LNM. In our study, all patients with central compartment LNM and/or lateral LNM were included, and the results showed that there were no significant differences in gender and multifocality between PTC with and without cervical LNM. The number of male patients (n = 5) had PTC with central cervical LNM was even less than female (n = 19).

Modern high resolution ultrasound (US) is regarded as the first choice for the preoperative diagnosis of PTC. Recent studies showed that US features may be also useful in predicting the biological behaviors of PTC. As the tumor size on ultrasound images is the most available preoperative parameter, someone reported^26^ that the maximum diameter of tumor was associated with a significantly increased risk of cervical LNM. Our results was in accord with this opinion, and the optimal cut-off value of tumor size obtained based on the ROC curve was 0.95 cm (AUC = 0.72). In the study of Nam *et al*.^27^, PTCs with more aggressive biological behaviors (LNM, extrathyroidal extension, and advanced stage) were defined as those showing at least one accepted ultrasonographic criterion: taller than wide shape, marked hypoechogenicity, microcalcifications, and infiltrative borders. In this study, we found that heterogeneous echogenicity, irregular shape, ill-defined margin, microcalcifications and ECE on US were more common in PTC with cervical LNM.

ECE is regarded as an important predictive factor of poor survival rate^28^. Patients with encapsulated carcinoma usually did not show distant metastasis and exhibited an indolent biologic behavior^29^. Thus, preoperative assessment of ECE would be of great value for the further clinical management. However, several studies recommended multi-slice computed tomography (MSCT) or magnetic resonance imaging (MRI) to evaluate ECE in PTC, since limitations still exist in US preoperative investigation^30, 31^. Recent studies utilized CEUS as a novel tool to detect ECE in PTC because of its microvascular imaging of capsular of thyroid lobe. In this study, ECE on CEUS was significantly different between PTCs with and without LNM (*P* = 0.000) and was demonstrated better than on US (81.48% vs 46.30%), furthermore, internal heterogeneous low-enhancement and peripheral irregular low-enhancement rings were mainly found in PTCs with LNM. Heterogeneous low-enhancement pattern^32, 33^ was not only regarded as a malignant parameter in the differential diagnosis of thyroid nodules in many reports (the mean microvessel density counts in PTC were less than in benign nodules such as nodular goiter and adenoma according to their pathological analysis), but also may be as an aggressive feature appearing more frequently in PTCs with cervical LNM than other kinds of internal enhancement patterns according to our clinical practice. Certainly, according to the previous studies^34, 35^, the degree of enhancement on CEUS had relations to the size of lesions. Most patients with PTC tend to show a low enhancement pattern when nodules are smaller than 1 mm, show an iso-enhancement pattern when nodules are 10–20 mm, and show a high-enhancement pattern when nodules are greater than 20 mm. Low enhancement may be caused by the small or immature vascular network in microcarcinomas. When the cancer grows quickly, various neovasculars start to form under the introduction of multiple angiogenic factors to meet the needs of fast growth. With rich and complicated vascular network, high-enhancement was commonly observed in large PTC. Meanwhile, for the growth heterogeneity and neovascular damage by malignancy infiltration, perfusion defect within lesions were often observed with the increase of lesion diameter, which could cause the heterogeneous enhancement features in thyroid tumor. In this study, 87.18% (136/156) PTCs were less than 20 mm, which may be the reason of why they mainly displayed internal low-enhancement patterns. Nowadays, malignant tumors with the size of over 20 mm are unusual, since many small suspicious nodules could be early detected by the periodic physical examination in our country. Therefore, internal heterogeneous low-enhancement pattern is still an important characteristic for malignancy and the risk of LNM. The ultrasound features of adjacent tissue surrounding the PTC correlated to the pathological changes caused by the invasive growth of tumor. A total of 39 tumors had peripheral rings during CEUS examination in this group, and they were regular no-enhancement ring, irregular high-enhancement ring and irregular no-enhancement ring. All these three types of peripheral rings could be found in the PTCs without cervical LNM (*P* > 0.05), whereas irregular no-enhancement ring was the most common type in the PTC with cervical LNM (*P* < 0.05). The adjacent thyroid tissue could be invaded by cancer, which might cause the appearance of the peripheral irregular no-enhancement ring as a result of the interstitial edema and fibrosis, hyaline degeneration or necrosis^24, 25^. Peripheral ring was not a common feature of PTC, but the irregular no-enhancement ring may predict the risk of cervical LNM comparing with other three types of peripheral rings (*P* = 0.04).

MMPs have been divided into collagenases, gelatinases, stromelysins and matrilysins. MMP-9, a member of the gelatinase group, not only readily digests denatured collagens and gelatins, but also plays a particular role in angiogenesis since it increases the bioavailability of proangiogenic factors^34, 35^. Our results showed that the intense expression of MMP-9 was mainly found in patients suffering from PTC with cervical LNM. The immunohistochemistry of MMP-9 can be easily performed on tissue sample by biopsy. Therefore, the conventional US and CEUS features in combination with MMP-9 expression may provide useful help for preoperative predicting the risk of cervical LNM from PTC.

Our study also had some limitations: A small number of tumors with peripheral rings on CEUS were included in this study because of random selection. Whether peripheral rings of PTC could predict the presence of cervical LNM should be confirmed in further long-term follow-up studies with a large sample.

In conclusion, tumor size ≥0.95 cm, ill-defined margin, internal heterogeneous low-enhancement and ECE detected in preoperative conventional US and CEUS findings were significantly associated with cervical LNM of PTC. Irregular no-enhancement rings were more commonly found in PTCs with cervical LNM comparing with other types peripheral rings. Besides, the intense expression of MMP-9 is also an important marker for the cervical LNM. Thus, the combination of conventional US, CEUS features and MMP-9 expression could serve as an effective tool for predicting the risk of cervical LNM from PTC.
